# Pennsylvania

**Published:** 1895-06

**Authors:** 


### PENNSYLVANIA.

The bill to establish a State Board of Veterinary Medical Examiners passed the Senate on May 7, 1895, with but few dissenting votes, and Governor Hastings signed it. This Act goes into effect June 1st. The Board will meet to organize in September next. The Governor has not yet announced his appointments.

Through the untiring efforts of Dr. Leonard Pearson, President of the Pennsylvania State Veterinary Medical Association, the bill establishing a Live-stock Sanitary Board passed the Senate on May 15, 1895. Governor Hastings signed the bill on May 21st, and its provisions will go into effect on June 1, 1895.

The bill to legalize dehorning of cattle in Pennsylvania was temporarily halted in its passage by the votes of the legislators of the cities and towns, under the strong influence and pressure of the Societies for the Prevention of Cruelty to Animals. The bill was afterward reconsidered, and passed the House, and it looks as if it would secure favorable consideration in the Senate and the approval of the Governor.

One of the bills before the present Legislature in Pennsylvania was designed to compel all milk-dealers selling skim-milk to so letter all the cans from which the same was delivered or sold. It met defeat in the House.

